# Fatigue in Multiple Sclerosis Compared to Stroke

**DOI:** 10.3389/fneur.2015.00116

**Published:** 2015-05-26

**Authors:** Claudia Lukoschek, Annette Sterr, Dolores Claros-Salinas, Rolf Gütler, Christian Dettmers

**Affiliations:** ^1^Kliniken Schmieder Konstanz, Konstanz, Germany; ^2^University of Surrey, Guildford, UK; ^3^University of Freiburg, Freiburg im Breisgau, Germany; ^4^Department of Neurology, University of São Paulo, São Paulo, Brazil; ^5^Lurija Institute, Kliniken Schmieder Allensbach, Allensbach, Germany; ^6^Department of Psychology, University of Konstanz, Konstanz, Germany

**Keywords:** assessment, fatigue, multiple sclerosis, questionnaire, SF-36, stroke, vitality

## Abstract

**Objectives:**

Fatigue is typically associated with multiple sclerosis (MS), but recent studies suggest that it is also a problem for patients with stroke. While a direct comparison of fatigue in, e.g., Stroke and MS is desirable, it is presently not easily possible because of different definitions and assessment tools used for the two conditions. In the present study, we therefore assessed fatigue in Stroke and MS using a generic, not disease-specific instrument to allow transdiagnostic comparison.

**Method:**

A total of 137 patients with MS and 102 patients with *chronic* stroke completed the SF-36, a generic questionnaire assessing health related quality of life. Fatigue was measured through the vitality scale of the SF-36. The vitality scale consists of two positive items (“lot of energy,” “full of life”) and two negative ones (“worn out,” “tired”). The two negative ones were scaled in reverse order. The vitality scale has been recommended as reciprocal index of fatigue.

**Results:**

Normalized vitality scores in MS (35.3) and stroke (42.1) were clearly lower than published reference values from the SF-36 in age-matched healthy controls. The sum score of the vitality items was lower in MS than in stroke patients. This difference could not be explained by age, gender, or the Physical Functioning Scale of the SF-36. Both patient groups showed no positive correlation between fatigue and physical functioning. Fatigue – as determined with the vitality scale of the SF-36 – correlated with the estimated working capacity in MS patients, but not in stroke patients.

**Conclusion:**

These findings confirm high fatigue in MS and stroke patients with higher values in MS. Fatigue has a higher impact on working capacity in MS than in stroke. Fatigue in both patient groups is not a direct consequent of physical functioning/impairment. Vitality score of the SF-36 is a suitable transdiagnostic measure for the assessment of fatigue in stroke and MS.

## Introduction

Fatigue is a prominent and frequent symptom in multiple sclerosis (MS), and affects 60–90% of patients (1, 2). Fatigue is often experienced as the most disabling and limiting symptom, and greatly contributes to the degradation of general well-being, quality of life, and social participation (3, 4). Moreover, the impact of fatigue in the workplace can be severe and frequently triggers early retirement, even in the early phase of the disease (5). In contrast to the importance of fatigue for patients, treatment options are limited and efficacy varies substantively across patients (6) (see also Khan et al., this special issue). Understanding and distinguishing different pathophysiological mechanisms might improve individually tailored treatment options.

While fatigue is most prominent in MS, it is also observed in other conditions. This is particularly for patients with Stroke, where fatigue has been identified as “a major yet neglected issue” (7). This perspective has spearheaded more research in this arena (8–10), but the characteristics of fatigue in stroke have yet to be fully determined. It is further unclear to what extent fatigue in MS and Stroke share similarities in their impact on the individual, and whether fatigue is equally prevalent in the two conditions.

Because fatigue is by far best characterized in MS, benchmarking fatigue characteristics of other conditions against MS is important. However, such comparisons are challenging because the majority of assessment instruments, such as the Fatigue Severity Scale (11) and the Fatigue Scale for Motor and Cognitive Functions (FSMC) (12), have been specifically developed for MS, and might therefore not be equally sensitive in other neurological conditions. Moreover, a recent review on fatigue measures in neurological conditions concluded that the FSMC and the Unidimensional Fatigue Impact Scale (13, 14) are best suited for measuring fatigue in MS, while the Profile of Mood States Fatigue subscale (POMS-F) is the optimal measure for stroke (15).

If fatigue characteristics and fatigue prevalence are to be compared across neurological conditions, it is necessary to use a generic, disease-unspecific measure, which allows the transdiagnostic comparison of fatigue prevalence. Such a generic measure has been derived from the vitality subscale of the short form SF-36 (15). The SF-36 is a well-validated and accepted measure of health, which is used in a wide range of health care settings and research (16). Its vitality subscale has already been used to assess fatigue in patients with myocardial infarction (17). The present study therefore used the vitality subscale to contrast fatigue in 137 MS and 102 Stroke patients. Based on the prevailing notion that the fatigue affects the majority of MS patients, we predicted a more severe manifestation and a higher impact on working capacity in MS compared to Stroke.

## Materials and Methods

### Participants

Data from 137 patients with MS (aged 47.3 ± 8.8, 51 males) and 102 patients with chronic stroke (aged 54.3 ± 12.0, 58 males), admitted to the hospital between January 2011 and March 2012, were included in the study. The data were retrospectively extracted from the database of the Kliniken Schmieder Konstanz, a specialized inpatient rehabilitation center in southern Germany. Kliniken Schmieder provides care for a wide range of neurological conditions but the largest patient groups comprise MS (800 patients per year), subacute, and chronic stroke (about 300 stroke patients per year). Patients typically stay in the clinic for 3–6 weeks. At the beginning of the stay, every patient completes the SF-36, a health related, generic questionnaire (16). All participants had a Barthel Index of >70 (qualifying for “Phase D” in the German rehabilitation system), and were able to independently exercise personal care.

For MS, the inclusion criteria comprised the confirmed diagnosis of MS, based on the McDonald criteria (18), for 12 months or longer. No further selection criteria were employed. The inclusion criteria for stroke encompassed hemorraghic or ischemic stroke which had occurred at least 12 months prior to testing. Transient ischemic attack (TIA) was not accepted as inclusion criterion. For both groups, exclusion criteria included (1) other neurological disorders such as head trauma, M. Parkinson, brain tumor, neuromuscular disorder, (2) history of psychiatric disorders, (3) major depression, and (4) cancer. In order to evaluate and compare the degree of impairment in both patient groups, the Physical Functioning Scale of the SF-36 and the participants’ retirement/employment and insurance status (for details, see next paragraph) were analyzed.

### Assessment

The SF-36 [German translation, version 1, (16)] was applied to all patients within 2 days of admittance to Kliniken Schmieder. The SF-36 is a psychometrically well-characterized (19) and widely used questionnaire to assess functional health and well-being. It contains 36 questions, which cover the following eight domains: vitality, physical functioning, bodily pain, general health perceptions, role physical functioning, role emotional, social functioning, and mental health. Scores on each item range from 0 to 100, with higher scores reflecting better functioning.

Fatigue was measured through the scores of the vitality domain (items 9a: “Did you feel full of life,” 9e: “Did you have a lot of energy,” 9g: “Did you feel worn out,” and 9i: “Did you feel tired”). These items are rated on a six-step Likert scale, and assigned values between 1 and 6. Because items 9a and 9e are positively scored, the respective raw scores were reversed prior to the transformation into standardized scores [transformed score = 100 × (raw value−minimal value)/range]. The average vitality (VT) score was calculated as the mean standardized scores of the VT items 9a, e, g, and i.

The level of physical disability was measured through the Physical Functioning Scale of the SF-36. This scale comprises 10 items (3a:vigorous activities, 3b: moderate activities, 3c: lift, carry groceries, 3d: climb several flights, 3e: climb one flight, 3f: bend, kneel, 3g: walk a mile, 3h: walk several blocks, 3i: walk one block, 3j: grooming and bathing). Responses are categorized according to the following options on a three-step Likert scale (1 = strongly impaired, 2 = moderately impaired, and 3 = not at all impaired), and transformed into standard scores ranging from 0 to 100 as described above.

Unfortunately at the time of admittance, we did not apply a standardized stroke scale for our patients like the NIH Stroke Scale or the Modified Ranking Scale (mRS) to describe characteristics of our patient population. But even if we had done so, it would have been difficult to compare these characteristics to MS patients, which are measured or scaled with different tools, most often with the Expanded Disability Status Scale (EDSS, see below).

In order to compare the handicap in both patient groups, the categorization of their working capacity assessment was taken from the discharge letters. In agreement with the work capacity classification system of German pension funds, the capacity for full time is defined as ≥6 h a day and part time as 3 to <6 h a day. A working capacity of <3 h a day corresponds to retirement. The working capacity is a medical prognostic judgment of the degree to which patients will be able to work after finishing sick leave. This judgment is independent of the actual employment status (i.e., all potentially eligible patients receive this judgment whether they are in employment or not). This categorization is not developed as a research tool, but is well standardized and affords excellent socioeconomic validity. It is also not specific for one diagnostic group, but allows for transdiagnostic comparison of restrictions in the working field.

The party covering the cost of the rehabilitation (pension funds in case of preserved working capacity and health insurance company in case of retirement) was determined. The working capacity measure is used uniformly across the range of health conditions and therefore provides a comparable real-world index of the capacity to work in both groups. Vitality and Physical Functioning were calculated for each working capacity category for both groups (Table 3).

The EDSS is documented in patients with MS. EDSS represents a common scale to quantify disability in MS patients, ranging from 0 to 10. Zero means no symptoms, 10 means dead due to MS. It is commonly used in clinical studies to characterize MS patients, and was therefore included in this study. The measure, however, is not meaningful to apply in patients with stroke, and is therefore reported for MS only.

### Statistics

Statistical analysis was conducted with SPSS version 19. Normal distribution of the variables was investigated using the Kolmogoroff–Smirnov test. Homogenous distribution of variances was confirmed by the Levene-test. Independent *t*-test was used to determine differences between vitality scores in stroke and MS patients. An ANOVA was calculated to look for interaction between diagnostic group and fatigue. A Pearson correlation was performed to analyze the correlation between fatigue and physical functioning. An ANCOVA was applied to investigate whether the difference between both patient groups was independent of age, sex, and physical functioning.

## Results

### Patients

The final sample comprised 102 patients with chronic stroke (mean age 54.3 ± 12.0 years) and 137 patients with MS (mean age 47.3 ± 8.8 years). Age was significantly different in both groups (*t*[177] = −5.02; *p* = 0.0005; η^2^ = 0.096). The gender balance in the two patient groups was different with 62.8% females in the MS group and 43.1% in the stroke group (χ^2^ [1] = 9.1; *p* = 0.003). In addition, scores on the Physical Functioning subscale of the SF-36 indicated significantly greater levels of disability in the MS group (17.8 ± 5.0) than in the stroke group (20.1 ± 5.6; *t*[183] = −3.2, *p* = 0.002). The EDSS – a scale developed for MS patients and not applicable in stroke patients – indicated a score of 4 reflecting moderate disability (3 refers to the border between light and moderate disability, 6 means depended on a walking aid to walk 100 m without rest). Although great care was taken only to include chronic stroke patients, time since onset of symptoms was longer in MS patients (15.5 years ± 9.3) than in stroke patients (5.2 years ± 6.0) due to its natural and chronic course.

Analysis of the employment status revealed that 70% of MS patients were funded by the pension fund compared to 76.5% in the stroke group (Table 1). These are the patients still working and those still under consideration for returning to work by the pension fund. At the time of discharge, almost 50% in both groups (46% of MS and 50% of stroke patients) were categorized as qualifying for a full time job. Almost 40% of the MS patients fell in the category for part-time work compared to 25% of stroke patients. In the stroke group, more patients had reached the status of being unable to work (25%) compared to the MS patients (14%) (Table 1).

**Table 1:** **Demographics and patients’ characteristics**.

	Patients with MS	Patients with stroke	Sign. level
*N*	137	102	
Female	63%	43%	<0.05
Mean age (range)	47.3 (20–69)	54.3 (21–80)	<0.05
Mean EDSS (SD)	4.1 (1.6)	Not applicable	
Range	0–8		
Years since onset, mean (SD)	15.5 (9.3)	5.2 (6.0); 1–33.6	<0.05
Range	1–49		
Party paying the rehabilitation
Pension fund (%)	70	76.5	
Health insurance company (%)	30	23.5	
Estimated working capacity			
>6 h	46.2%	50.8%	
3–6 h	39.5%	24.6%	
<3 h	14.3%	24.6%	
Physical Functioning Scale from SF-36 (SD)	17.8 (5.0)	20.1 (5.6)	<0.05

*EDSS, Expanded Disability Status Scale*.

*Paying party: as long as the pension fund pays for rehabilitation, the client is still in the category of being or becoming potentially able to work. “Estimated working capacity” displays the number of full-time (>6 h) and part-time (3–6 h) workers as well as the number of those being unable to work anymore. The paying party and the estimated working capacity indicate that disability in both groups was similar*.

### Fatigue score

Mean values on the normalized vitality subscale of the SF-36 were 35.4 ± 12.1 in MS patients and 42.1 ± 12.7 in stroke patients (compare Table 2). These means are well below the vitality data available through the German Health Survey 1998 (20).

**Table 2:** **Normalized Vitality scores of the vitality subscale of the SF-36 from the present investigation compared to normal values from the German Health Survey 1998 (20)**.

Patients/reference group	Mean	SD	Comment Original publication
MS	35.4	12.1	Present data
Stroke	42.1	12.7	Present data
Male, age 40–49	64.2	16.3	German Health Survey 1998 (20) (*N* = 6964 participants, age 18–80)
Male, age 50–59	61.5	18.1	German Health Survey 1998 (20)
Female, age 40–49	57.4	18.8	German Health Survey 1998 (20)
Female, age 50–59	57.7	18.8	German Health Survey 1998 (20)

Statistical analysis of the vitality scores further suggested a highly significant group difference (*F* = 7.49; *p* = 0.007), reflecting higher levels of fatigue in MS than Stroke. This group difference remained when age, sex, and Physical Functioning were factored in as covariates (*F*[1,236] = 4.59; *p* = 0.033; η^2^ = 0.02 for age; *F*[1,236] = 5.96; *p* = 0.015; η^2^ = 0.03 for sex; *F*[1,213] = 9.19; *p* = 0.003; η^2^ = 0.04 for Physical Functioning). Both groups did not show positive correlations between fatigue and physical functioning as determined by question three of the SF-36 (Pearson correlation).

Separate calculation of the vitality scores for each category of estimated working capacity further revealed that vitality was closely related to working capacity in MS patients (*r* = 0.25; *p* = 0.02; Pearson correlation), but not in stroke patients. By contrast, Physical Functioning is associated to the estimated working capacity in stroke patients but not in MS patients. These data are summarized in Table 3.

**Table 3:** **Vitality and Physical Functioning in relation to working capacity in MS and stroke patients**.

Estimated working capacity	Vitality			10-item Physical Functioning		
	*N*	Mean (SD; range)	Median	*N*	Mean (SD; range)	Median
**Patients with MS**						
>6 h	43	38.2 (15.8; 5–75)	40	41	52.1 (26.1; 5–100)	50
3–6 h	36	32.8 (21.3; 0–80)	30	33	30.8 (26.9; 0–100)	35
<3 h	13	25.0 (10.0; 5–40)	25	11	30.0 (13.2; 5–45)	35
**Patients with stroke**						
>6 h	32	44.8 (18.4; 15–85)	42.5	32	64.2 (24.1; 15–100)	67.5
3–6 h	15	31.7 (13.2; 10–60)	30	14	56.8 (23.7; 20–95)	60
<3 h	16	41.6 (22.4; 0–80)	40	16	37.2 (31.0; 0–100)	30

*Vitality appears to be proportional to working capacity in MS, but not in stroke patients. Working capacity had been estimated from the medical doctor at the time of discharge only in those patients, whose rehabilitation had been paid from the pension funds (92 patients with MS, 62 patients with stroke)*.

## Discussion

The present study used the vitality score derived from the SF-36 as an index of fatigue. The data shows that the vitality scores derived in patients with MS and stroke are lower than normal values of population based studies. This suggests that patients with stroke and patients with MS suffer greater fatigue than their healthy peers. The data further suggests that fatigue is a substantive issue in both patient groups.

This study allows for a direct comparison between the SF-36 vitality score as a proxy for fatigue in stroke and MS patients. The SF-36 is a widely evaluated generic patient-assessed health outcome measure (21). The generic character of the questionnaire enables transdiagnostic comparison of patients with different conditions. In the present study, the comparison was conducted for fatigue and showed that fatigue in stroke patients falls within a similar range as in MS patients. This is an important finding since fatigue, recognized as a major issue in clinical practice, is much less recognized in patients with stroke.

However, fatigue in MS patients is still higher than in stroke patients. This might have been expected at least from health professionals and cares dealing with MS patients.

It appears remarkable to us that there is an association of fatigue and working capacity in MS patients, but that there is not such an association in stroke patients. In our view, this confirms the clinical impression that fatigue has a high clinical impact on MS patients, but less so in stroke patients. In other words: the close association of fatigue with working capacity in MS patients suggests that fatigue directly affects working capacity in MS patients. This is not the case in stroke patients; here, working capacity is more related to Physical Functioning.

The data confirm that fatigue is more prominent in MS than stroke. This is a very important finding; since to our knowledge, only few publications have investigated fatigue in MS and stroke patients using the same assessment tool. Naess et al. obtained the Nottingham Health Profile in 191 ischemic stroke patients and compared it to 337 MS patients (22). It was concluded that stroke patients often report pain and problems with sleepiness, while MS patients often report more problems with fatigue. Using alertness as a marker of fatigue in MS and stroke patients, Claros-Salinas et al. (23) demonstrated an increase in reaction time during the course of the day, highlighting the similarity between these two patient groups. At the same time, the decline of performance in MS patients appeared slightly greater than in stroke suggesting more pronounced fatigue in MS patients compared to stroke patients. In contrast, no decline was found for age matched controls. Mills and colleagues further developed a new fatigue index for MS patients (24), and validated the instrument for assessment of fatigue in stroke patients (25). They concluded that “post-stroke fatigue appeared to be qualitatively similar to that of MS fatigue, including, for example, features associated with physical and cognitive aspects” (25). The present study therefore provides further evidence that fatigue is an important symptom in the chronic phase of stroke. Whether the pathophysiological mechanisms underlying fatigue in stroke and MS are similar or not, however, it needs to be determined in future research.

Importantly, neither patient group showed a positive association between the vitality subscale and the Physical Function subscale. It is remarkable that the degree of fatigue reported here cannot be explained by a simple effect of limitations in physical functioning. Our results suggest that fatigue is not a consequence of the accumulation of tissue damage. This is in line with recent observations showing that the Motricity index as well as the Stroke Impact Scale are not predictive of fatigue (9). Similarly, structural computer tomography variables (atrophy, white matter lesions, or previous vascular lesions) were not associated with fatigue at 1 month (26). Previous investigations could not confirm a significant correlation between EDSS and fatigue (27, 28). This stands in contrast to other studies reporting a correlation between fatigability and motor (“pyramidal”) involvement and disability (29, 30). While it may be plausible that patients have more fatigue in the advanced stage, there seems to be no *close* correlation between physical impairment and fatigue. Our data confirm that fatigue is not a direct consequence of physical impairment in MS or stroke.

### Study limitations

While the use of a general health questionnaire has the advantage of being applicable in two different patient groups, it has the obvious disadvantage that it is not a precise instrument, which can capture fatigue in all its facets. In other words, the benefit of comparability comes at the cost of accuracy with which fatigue is assessed. Although stroke and MS patients rated their vitality in a similar range, confronting patients with a more elaborate questionnaire or *measuring* reaction time as a surrogate marker of fatigue before and after a cognitive challenging task (31) might provoke different results and might show larger discrepancies between stroke and MS patients. Although fatigue falls in a similar range in both entities, in our opinion the question remains, whether or not fatigue in stroke patients is as disabling as in MS patients. We assume that the vitality score is not elaborate enough to capture the complete phenomenon of fatigue and to compare the disabling impact of fatigue in both diseases. Nevertheless, the SF-36 is widely applied, easy to handle, and allows for transdiagnostic comparison between different patient groups.

Another limitation might be the selection of our MS and stroke patients. We did not include severely affected stroke patients, who require further assistance in daily activities. Neither did we include stroke patients with a very good prognosis, who do not require any rehabilitation. The present findings might therefore not be generalizable to the whole range of longer term outcome present in stroke survivors.

## Conflict of Interest Statement

The authors declare that the research was conducted in the absence of any commercial or financial relationships that could be construed as a potential conflict of interest.
